# *In vivo* models in breast cancer research: progress, challenges and future directions

**DOI:** 10.1242/dmm.028274

**Published:** 2017-04-01

**Authors:** Ingunn Holen, Valerie Speirs, Bethny Morrissey, Karen Blyth

**Affiliations:** 1 Academic Unit of Clinical Oncology, University of Sheffield, Sheffield S10 2RX, UK; 2 Leeds Institute of Cancer and Pathology, University of Leeds, Leeds LS9 7TF, UK; 3 Cancer Research UK Beatson Institute, Glasgow G61 1BD, UK

**Keywords:** Breast cancer, Mouse models, GEMM, PDX, CDX, SEARCHBreast, EurOPDX

## Abstract

Research using animal model systems has been instrumental in delivering improved therapies for breast cancer, as well as in generating new insights into the mechanisms that underpin development of the disease. A large number of different models are now available, reflecting different types and stages of the disease; choosing which one to use depends on the specific research question(s) to be investigated. Based on presentations and discussions from leading experts who attended a recent workshop focused on *in vivo* models of breast cancer, this article provides a perspective on the many varied uses of these models in breast cancer research, their strengths, associated challenges and future directions. Among the questions discussed were: how well do models represent the different stages of human disease; how can we model the involvement of the human immune system and microenvironment in breast cancer; what are the appropriate models of metastatic disease; can we use models to carry out preclinical drug trials and identify pathways responsible for drug resistance; and what are the limitations of patient-derived xenograft models? We briefly outline the areas where the existing breast cancer models require improvement in light of the increased understanding of the disease process, reflecting the drive towards more personalised therapies and identification of mechanisms of drug resistance.

## Abstract (precis)

**Summary:** This Review provides a summary of the many varied uses of mouse models in breast cancer research focusing on their strengths, challenges and future directions.

## Introduction

Clinical management of breast cancer has improved significantly over the past 30 years, with almost 87% of women surviving their diagnosis for at least 5 years compared with only 53% of those diagnosed in the early 1970s (cancerresearchuk.org; accessed December 2016). Nonetheless, breast cancer remains the leading cause of cancer-related female death worldwide with more than half a million women succumbing to the disease annually (Torre et al., 2016) including around 11,500 in the UK (breastcancernow.org; accessed December 2016). One of the reasons for this is that breast cancer is not a single disease entity (Curtis et al., 2012) and ‘one size’ does not ‘fit all’ for clinical management, treatment nor, as discussed here, modelling of the disease. Breast cancer is treated based on the receptor status of the tumour, specifically oestrogen receptor (ER), progesterone receptor (PR) and human epidermal growth factor receptor-2 (HER2), and the main molecular subtypes are termed Luminal A (ER/PR-positive); Luminal B (ER/PR-positive, higher histological grade than Luminal A); HER2-positive; and triple-negative (ER/PR/HER2-negative) (Cardiff and Kenney, 2011). Tailored therapies have led to considerable success in treating some breast cancers, such as hormone therapies (e.g. tamoxifen, and inhibitors of the enzyme aromatase, involved in oestrogen synthesis) for ER-positive disease, and trastuzumab (Herceptin) for HER2-positive breast cancer; however, drug resistance to these regimes is common (Osborne and Schiff, 2011; Palmieri et al., 2014; Luque-Cabal et al., 2016). Furthermore, there is still no good targeted therapy for triple-negative breast cancer, which is one of the more aggressive subtypes of the disease (Kalimutho et al., 2015; Gu et al., 2016). In addition, whilst primary breast cancer is highly treatable [80-99% of women diagnosed with stage I/II breast cancer survive to 5 years; (cancerresearchuk.org; wcrf.org)], there is no cure currently available for metastatic breast cancer, which affects an estimated 40% of UK patients (breastcancernow.org) and likely accounts for the decline in survival rate to 65% at 20 years post-diagnosis. Genetic sequencing endeavours have identified many of the mutations implicated in breast cancer (Nik-Zainal et al., 2016), which may lead to the development of new therapeutic options; however, the functional role of these alterations in the different subtypes has still to be confirmed, and their distinct roles during disease progression, tumour heterogeneity and dormancy, clarified (Eccles et al., 2013).

**Fig. 1.**
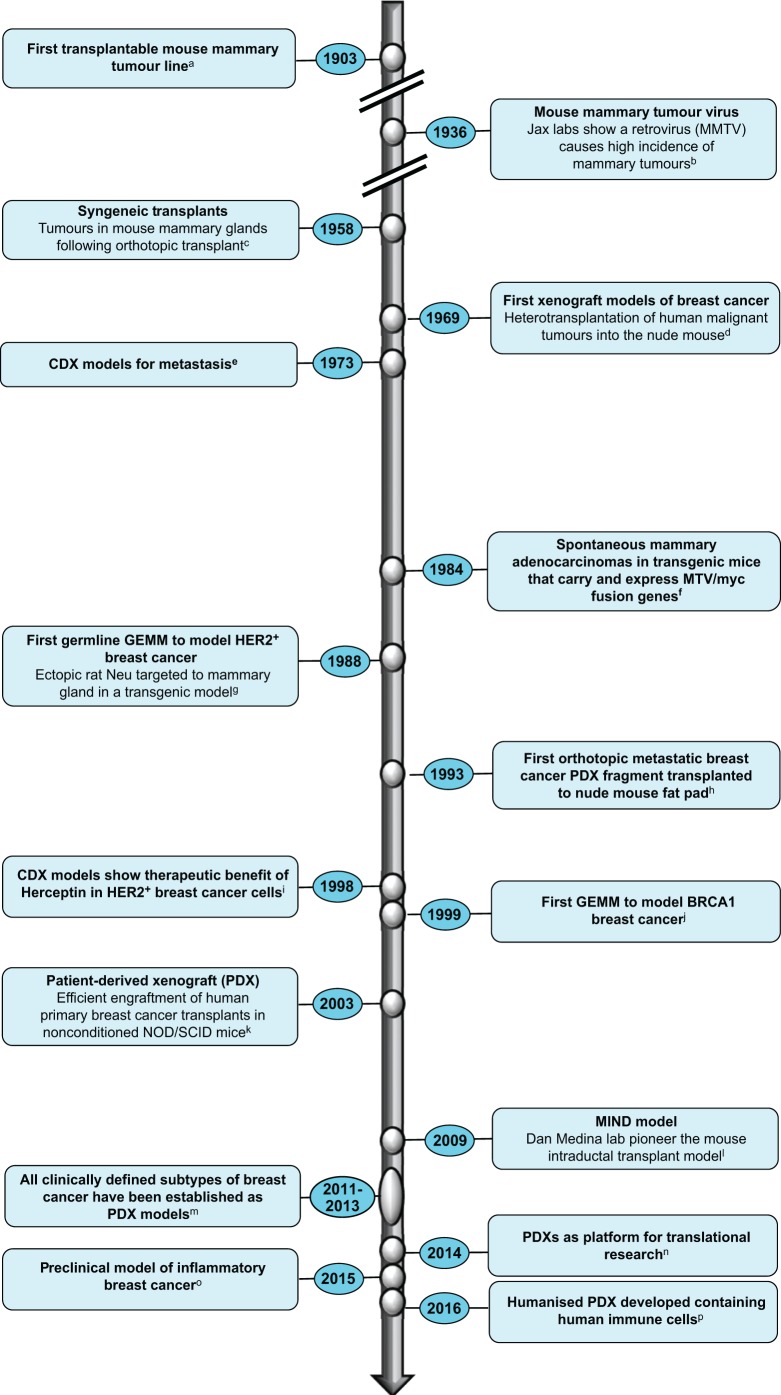
**Modelling breast cancer through the ages.** The figure depicts a timeline of key events or developments in the evolution of mouse models of breast cancer over the years. Lowercase letters denote the following references, to which the reader is referred for further reading on specific milestones: ^a^Cardiff and Kenney, 2011; ^b^Bittner, 1936; ^c^DeOme et al., 1959; ^d^Rygaard and Povsen, 2007; ^e^Fidler, 1973; ^f^Stewart et al., 1984; ^g^Muller et al., 1988; ^h^Fu et al., 1993; ^i^Baselga et al., 1998; ^j^Xu et al., 1999; ^k^Beckhove et al., 2003; ^l^Behbod et al., 2009; ^m^DeRose et al., 2011; ^n^Whittle et al., 2015; ^o^Wurth et al., 2015; ^p^https://www.jax.org/news-and-insights/jax-blog/2015/april/the-next-big-thing-in-cancer-modeling-patient-derived-xenografts-in-humaniz.

Scientists have capitalised on *in vivo* models as important research tools to study the pertinent questions in breast cancer research (Fig. 1). By ‘*in vivo*’ we refer to a living organism and we will restrict our discussions to the mouse, a physiologically relevant system in which to explore cancer initiation, invasion and metastasis, and which represents an essential step between *in vitro* systems and clinical studies. Researchers now have access to a broad range of mouse models, each with its own strengths and limitations (see overview in Table 1). A clear understanding of these parameters is paramount in order to choose the model best suited to address the specific research questions posed. SEARCHBreast, an organisation dedicated to sharing animal resources (see Box 1), hosted a workshop (searchbreast.org/workshop3.html) to showcase the contribution these complex models have made to breast cancer research highlighting recent developments and discussions on how shortfalls in existing models could be addressed in the foreseeable future. The workshop, open to all UK researchers with an interest in breast cancer, involved presentations and discussions from leading groups with broad expertise in utilising breast cancer mouse models in a variety of areas, from disease mechanisms to preclinical trials. In this workshop-inspired perspective, we highlight recent progress in mouse models of breast cancer, and discuss some of the outstanding issues researchers are grappling with in their pursuit of the best *in vivo* model for their research question. The article is not intended to give an exhaustive review of the vast literature but will give readers a current overview of the advantages, limitations and challenges that lie ahead for breast cancer researchers and especially those utilising, or wishing to adopt, mouse models to study this heterogeneous disease.

**Table 1.**
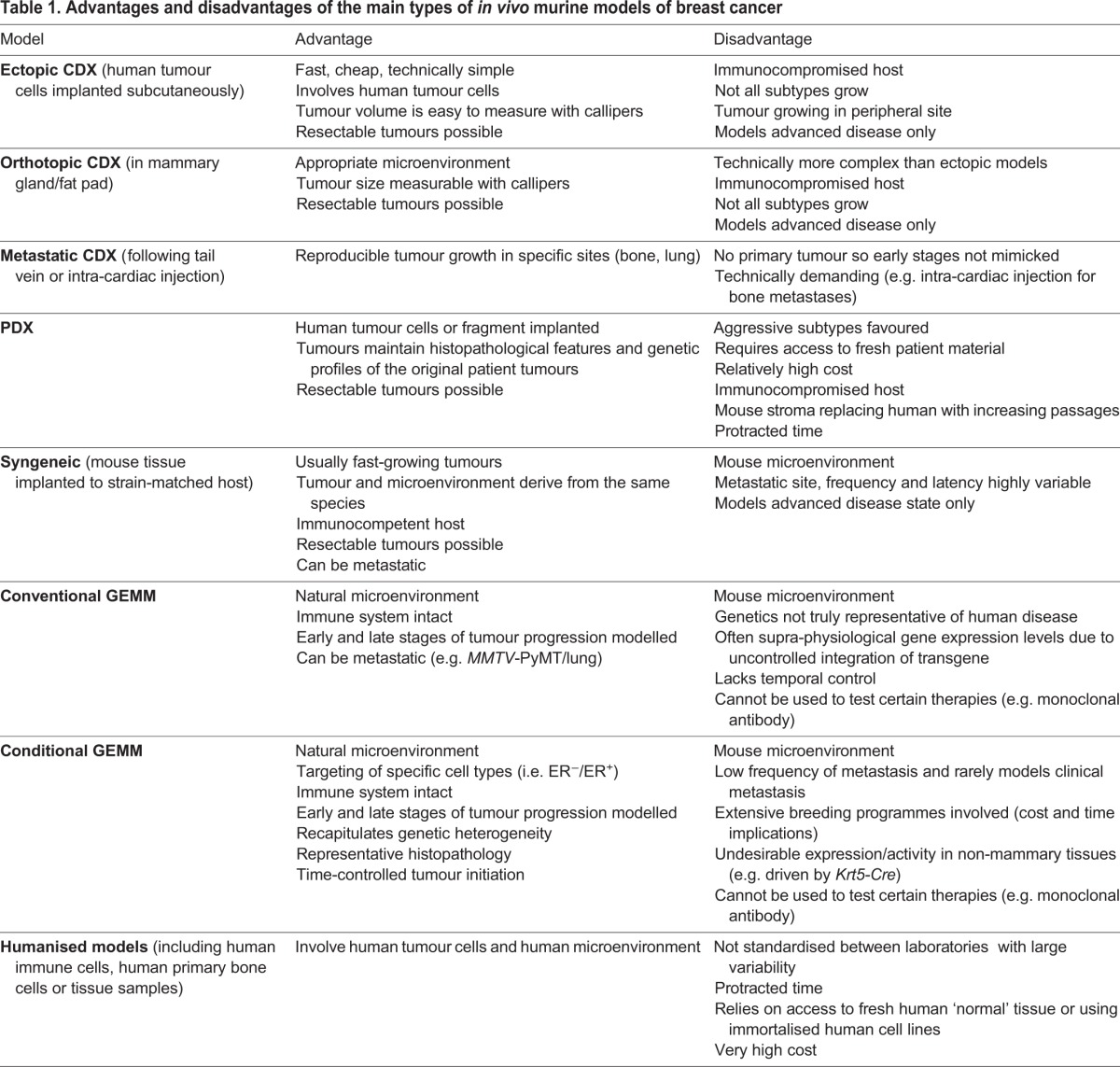
**Advantages and disadvantages of the main types of *in vivo* murine models of breast cancer**

**Box 1. SEARCHBreast and EurOPDX**

The workshop was hosted by SEARCHBreast (sharing experimental animal resources: coordinating holdings – Breast, www.searchbreast.org); a novel resource funded by the National Centre for the Replacement, Refinement and Reduction of Animals in Research (NC3Rs) to create a secure, searchable database of archived material derived from *in vivo* breast cancer models, which is available to share between researchers (Blyth et al., 2016). Use of this resource could allow new animal experiments to be avoided, or refined, ultimately reducing the number of animals used for research, as well as saving the time and money required to run *in vivo* experiments from scratch (Morrissey et al., 2016). At the heart of SEARCHBreast is the fact that many labs have surplus tissues from *in vivo* experiments left over at the end of a study, while other researchers would benefit from access to such material. SEARCHBreast is a no-cost mediator that brings together these collaborations. The web-based platform contains information on thousands of tissue samples that are available for immediate use following a simple online request. Samples are available free of charge from over 85 different mouse models, including GEMM, xenograft and patient-derived xenografts. With almost 250 members across the UK and internationally, SEARCHBreast is a large network of breast cancer researchers with collective experience with *in vivo*, *in vitro* and *in silico* models of breast cancer.

The EurOPDX consortium (www.europdx.eu), established from partners in 10 different European countries covering 16 academic institutions, is a network of cancer scientists and clinicians who have pioneered the standardisation of PDX models as clinically relevant models of human cancer. EurOPDX has created an extensive virtual collection of PDX models (>1500) encompassing many cancer types that have been genomically and histologically characterised in well-established laboratories. The aim of the consortia is to unify and harmonise the use of PDX models, which could lead to better resources for investigating predictive biomarkers and testing novel therapeutic approaches, and ultimately, personalised cancer treatment.

## Modelling breast cancer development, therapeutic targeting and drug resistance

One of the main challenges in developing *in vivo* models has been the increasing understanding of the many different subtypes of breast cancer (Perou et al., 2000; Sorlie et al., 2003; Curtis et al., 2012; Cancer Genome Atlas Network, 2012). Ideally, this complexity should be faithfully reflected in model systems. Although the field has access to a wide variety of mouse models that have evolved over the years (Fig. 1), certain models are much more commonly used than others and not all subtypes are represented. The main model systems and their application to date are briefly outlined below.

## Cell-line-derived xenografts

One of the simplest and therefore most commonly used model systems is based on engraftment of human cell lines (Neve et al., 2006) to immunocompromised animals [cell-derived xenografts (CDX)]. These have proven to be extremely useful for assessment of breast cancer genetics, biological processes, and to some degree, metastatic potential; but are limited by their reduced intra-tumoural heterogeneity and their poor record of predicting clinically effective therapies (Whittle et al., 2015 and references therein). In addition, the lines used are frequently derived from highly aggressive malignant tumours or plural effusions (fluids drained from lung metastasis) such as the frequently studied MDA-MB-231 line, making these less useful for modelling early events in the evolution of the primary tumour. Although well-characterised cell lines representing the common clinical subtypes – luminal A (e.g. MCF-7, T47D), luminal B (e.g. BT474, MDA-MB-361), HER2^+^ (e.g. SKBR3, HCC202) and triple negative (e.g. BT20, MDA-MB-231, MDA-MB-468) – have been extensively studied, not all can be established *in vivo* (Holliday and Speirs, 2011) and in particular there is a dearth of tumourigenic HER2^+^ lines. Furthermore, long-term growth *in vitro* can result in aberrant selection pressures.

CDX models represent a relatively homogenous mass of transformed breast epithelial cells and as such do not capture the heterogeneity of human breast tumours, which arise in a niche of cell types that have symbiotically evolved together (Hanahan and Weinberg, 2011). Indeed the use of CDX to model the native tumour microenvironment is difficult, particularly because of the necessity to use immunocompromised recipients lacking an effective immune system. The site of transplantation should also be considered. This can be straightforwardly achieved via subcutaneous injection (ectopic), or by implanting cells in the mouse mammary gland (orthotopic), which is more complicated. The diverse microenvironment of these sites, particularly the tumour vasculature, significantly impacts tumour growth rate (Fleming et al., 2010), drug delivery and therapeutic efficacy – something that should be considered when choosing a cell-line-based model for preclinical testing of novel agents and/or treatment schedules (Talmadge et al., 2007; Fung et al., 2015).

Finally, spontaneous metastasis from CDX models is rare, hampering studies of breast cancer metastasis using this type of model. However, a few characterised murine cell lines (e.g. 4T1) do metastasize in syngeneic models (involving transplant of mouse-derived cells to an independent strain-matched mouse), although these are currently a limited resource (Johnstone et al., 2015; Erler et al., 2009; Paschall and Liu, 2016). Otherwise, direct injection of breast cancer cells into ectopic sites (e.g. bloodstream, femur) can be used as an experimental metastasis assay.

## Patient-derived xenografts

Patient-derived xenografts (PDXs) – involving the transplantation of primary human cancer cells or tumour pieces into host mice – were developed to address the shortcomings of CDX, heralding hope for models with improved clinical relevance. Although transplantation of human tumour fragments into immunocompromised mice has a long history (reviewed in Hoffman, 2015), there is renewed interest in PDX models because of the preservation of many relevant features of the primary human tumour, including growth kinetics, histological features, behavioural characteristics (such as invasiveness and metastatic capacity) and most importantly, response to therapy (Marangoni et al., 2007; DeRose et al., 2011; Tentler et al., 2012; Hidalgo et al., 2014). Indeed, Ledford (2016) reported that the US National Cancer Institute (NCI) are planning to replace their NCI-60 cell line resource with PDX samples, which underscores the importance and acceptance of this resource.

### Use of PDX models to model disease subtypes and metastasis

Modelling luminal ER^+^ subtypes, which are found in over 70% of diagnosed individuals, making these the commonest form of human breast cancer, has been particularly challenging. This is partly because successful xenotransplantation selects for the most aggressive subtypes, hence biasing both CDX and PDX towards the triple-negative subtype (reviewed by Tentler et al., 2012; Cottu et al., 2012). The recent demonstration, however, that transplanting ER^+^ cells (derived from cell lines and primary patient material) directly into the mouse ductal epithelium rather than the fat pad (Behbod et al., 2009) can preserve the luminal/ER^+^ phenotype of these cells through avoiding activation of the TGFβ signalling pathway, is encouraging (Richard et al., 2016; Sflomos et al., 2016). Not only does this facilitate establishment of a less-aggressive breast cancer subtype, but more importantly maintains faithful representation of the naïve human disease and has been described as a ‘potential game-changer’ for preclinical modelling of ER^+^ breast cancer (Haricharan et al., 2016). Indeed, PDXs derived from ER^+^, HER2^+^ and triple-negative disease, representing the main clinical subtypes, have been described (Dobrolecki et al., 2016).

Importantly, encouraging results have been obtained in which PDX models effectively model metastasis, with recent protocols showing preservation of patterns of metastatic spread representative of the clinical situation (DeRose et al., 2013). However, as PDXs are most commonly generated at the point of breast cancer surgery, it is difficult to assess to what extent their capacity to form metastases recapitulate that of the patient without a follow-up period of at least 5 years. A number of studies have reported apparent discrepancies between the metastatic patterns of PDX compared with those of the patient from which they originate. The most common metastatic site in the PDX models are lungs and lymph nodes, whereas brain and bone metastases are rarely reported, despite occurring frequently in patients (Eyre et al., 2016; Whittle et al., 2015).

### PDX models for preclinical therapy testing

Evidence supporting the usefulness of PDX models in identifying clinically relevant treatment resistance mechanisms is amassing (ter Brugge et al., 2016). This has even been demonstrated in hard-to-model ER^+^ breast cancer, in which phosphoinositide 3-kinase (PI3K) pathway activation and outgrowth of stem cell populations, have been found during acquired resistance to anti-oestrogen therapies (Cottu et al., 2014; Simões et al., 2015). Key challenges that still need to be addressed, however, include the overall under-representation of ER^+^ tumours, as well as the aforementioned bias towards more aggressive tumours. Furthermore, such bias towards aggressive tumours may falsely infer therapeutic benefit (or indeed not) considering that less-aggressive cancers may not respond in the same way to therapy as those which are more proliferative and/or have different molecular alterations. Finally, the study of the tumour microenvironment is compromised both by intrinsic species differences (e.g. mouse fat pad) and the lack of immune cells in tolerant hosts. This latter point is notable considering the impact of immune-based therapies in the clinic (Varn et al., 2016) and is discussed below.

Some have mooted the possibility of PDX models as mouse ‘avatars’ for personalising treatment to individual patients; however, clinical decision-making is quick (weeks), compared with the time taken to establish a PDX and using it to test the response to different therapeutic regimens (months to years), so currently the potential of these models is limited to cohort-based preclinical studies. Regardless of these limitations, the wide acceptance of PDX models in the pharmaceutical industry is worthy of mention. There has been an explosion in the commercial sector in the use and availability of PDXs as the model of choice for translational research. Companies such as Champions Oncology and Crown Biosciences offer partnerships to run preclinical drug trials, while certain suppliers such as Horizon Discovery and The Jackson Laboratories make these models available to academic institutions. This is a fast-moving field and efforts are now being honed into producing humanised systems (see below).

## Genetically engineered mouse models

For addressing early events in the tumour process, genetically engineered mouse models (GEMMs) come into their own. In these models, spontaneous tumour initiation occurs within the correct microenvironment from an otherwise normal mammary cell. These may be simple oncogenic-driven transgenic mice, referred to here as conventional GEMM (e.g. MMTV-PyMT). One limitation to the conventional GEMM models is that not only are the regulatory sequences used to drive transgene expression not well-defined in terms of specific lineage/expression domains, but also, the specific oncogenes may not necessarily reflect those observed in human tumours. Nonetheless, these models continue to serve a purpose in breast cancer research (Blaas et al., 2016; Arun et al., 2016).

With these limitations in mind, the field has turned to more specific models emulating the genetics of human disease with spatial and temporal activation of oncogenes and deletion of tumour suppressors targeted to the mouse mammary gland (e.g. *Blg*-*Cre;Brca1^fl/fl^;p53^fl/fl^*). These conditional GEMMs use the Cre/*loxP* system in which a tissue-specific promoter drives expression of the bacterial enzyme Cre recombinase (e.g. *Blg-Cre*) within the mammary gland to elicit recombination of DNA between *loxP* sites (e.g. introduced into the coding region of tumour suppressors such as *p53* and *Brca1*). Lists of the many different breast cancer GEMMs (conventional and conditional) can be found in other recent reviews (Pfefferle et al., 2013; Borowsky, 2011; Menezes et al., 2014; Dabydeen and Furth, 2014; Greenow and Smalley, 2015; Ben-David et al., 2016).

### Recreating tumour pathology in GEMMs

Pathological profiling has been essential in disease classification, prognosis and disease management and remains the clinical basis of stratification, so it is important that as well as genetic complexity, the tumour pathology is faithfully recapitulated in genetic models (Blyth et al., 2012). This has worked well for some epithelial cancers (e.g. pancreas, colon); however, there is a concern that models of breast cancer do not always reflect the pathology of the human disease. Historical models of spontaneous and mouse mammary tumour virus (MMTV)-infected tumours (Fig. 1) do not share histological features of human tumours (Cardiff et al., 2004) while specific oncogenic models in the form of conventional GEMMs (e.g. *MMTV*-*Wnt1* and *MMTV*-PyMT) can demonstrate phenotypic similarities as well as disparities (Cardiff and Wellings, 1999). A consensus of medical and veterinary pathologists voiced caution over the interpretation of tumours derived from breast cancer GEMMs and advocated the inclusion of pathology expertise in any research team using these models (Cardiff et al., 2000). With an exponential rise in the number of breast cancer models now available, there has been a push to revisit these guidelines and a meeting of *in vivo* biologists and pathologists is planned for the near future. Meanwhile, publications (Munn et al., 1995; Cardiff et al., 2000; Ponzo et al., 2009; Cardiff, 2010; McCarthy et al., 2007; Fathers et al., 2010; Meyer et al., 2013; Melchor et al., 2014; Bao et al., 2015) and online tools (tvmouse.ucdavis.edu/pathology/) are available to aid in the comparative assessment of GEMM tumour pathology.

### Modelling breast cancer subtypes in genetic models

The mature mammary gland is principally composed of two epithelial lineages comprising the luminal and basal cells. At the simplest level, breast cancer heterogeneity arises from origins in these different cell types with unique and associated gene signatures in distinct breast cancer subtypes (Sorlie et al., 2001; Prat and Perou, 2011). Recreating these subtypes in GEMMs has been attempted by targeting a variety of oncogenic drivers to the different mammary lineages. To this end, keratin 14 promoter (*Krt14*)-*Cre* or keratin 5 promoter (*Krt5*)-*Cre* have been used to direct oncogenic events to the basal lineage; whey acidic protein promoter (*Wap*)-*Cre* and keratin 8 (*Krt8*)-*Cre* to target luminal cells, and β-lactoglobulin (*Blg*)-*Cre* and *Cited1*-*Cre* to specifically target ER^−^ and ER^+^ luminal cells, respectively. However, despite the use of lineage-specific promoters, a complete characterisation in the expression pattern of these Cre recombinases has not been done, and so ‘off-target’ and/or unexpected expression can confound studies. For example, *Krt14*-*Cre* can be active in the luminal as well as basal lineages (Jonkers et al., 2001; Regan et al., 2012), hampering complete separation of events in these populations. Furthermore, there is a lack of specificity in some Cre recombinases, such as with *Krt14*-*Cre*, which is expressed in skin and dental epithelium (Dassule et al., 2000), resulting in issues with skin tumours (Liu et al., 2007) or normal feeding capability, depending on the genetic changes introduced by that promoter. Caveats aside, genetic profiling has aligned GEMMs with specific molecular subtypes (Herschkowitz et al., 2007; Pfefferle et al., 2013; Hollern and Andrechek, 2014), highlighting the utility of genetic models in modelling the human disease.

### Usefulness of GEMMs in determining the cell of origin

The use of lineage-restricted GEMMs has been instrumental in studies to identify the cell of tumour origin (Lindeman and Visvader, 2010; Beck and Blanpain, 2013; Brooks et al., 2015; Koren and Bentires-Alj, 2015). A prime example of this was the demonstration that breast cancer 1 susceptibility gene (*Brca1*)-deficient breast cancers originated from luminal progenitors, contrary to the expectation that these were of basal stem cell origin (Molyneux et al., 2010; Lim et al., 2009). However, breast cancer is not just one disease and whether there are one or more different types of tumour-initiating cell remains to be determined. The origin of the normal post-natal mammary tissue itself (from either a multi-lineage basal mammary stem cell, or a lineage-restricted progenitor) has been a source of contention, and extensively explored using *in vivo* lineage tracing and transplantation models (Visvader and Stingl, 2014; Wuidart et al., 2016). Studying the tumour-initiating cell in genetic models is also hindered by the variable efficiencies of different reporters that are used to ‘tag’ cell populations and the specificity/off-target expression of Cre recombinase, as described above. Furthermore, the effect of the driver mutation on the targeted cell needs to be considered. *PIK3CA* (phosphatidylinositol 3-kinase, catalytic, alpha polypeptide), a common genetic driver of breast cancer, invokes mixed-lineage tumours even when expressed in unipotent progenitor cells, demonstrating plasticity (Van Keymeulen et al., 2015; Koren et al., 2015) as well as lineage switching (i.e. expression of *PIK3CA* in a basal-progenitor cell giving rise to luminal tumours).

### Preclinical testing in genetic models

Whilst targeted therapies have been a resounding success in breast cancer, relapse due to drug resistance remains a problem. GEMMs have been used to define the molecular mechanisms of drug resistance *in vivo* and have the advantage that pathway inactivation can be achieved either genetically and/or pharmacologically. For example, deregulation of cell cycle control (Goel et al., 2016) or loss of phosphatase and tensin homologue (PTEN) (Creedon et al., 2016) were shown to be important in conferring resistance to anti-HER2 therapies (e.g. trastuzumab). Of course, these mechanisms have to be shown to be relevant to the human disease; hence validation in primary patient material is essential. Towards this goal, *c-Myc* amplification in a genetic model of *PIK3CA*-related breast cancer was discovered to circumvent PI3K-targeted treatment, in agreement with the observation that high MYC levels aligned with mutation of *PIK3CA* in patient samples (Liu et al., 2011). Also, when searching for mechanisms of resistance to poly(ADP ribose) polymerase (PARP) inhibitors in *BRCA1*-associated disease, downregulation of the tumour suppressor p53-binding protein 1 (*53BP1*) and its downstream effector *MAD2L2* (MAD2 mitotic arrest deficient-like 2; previously known as *REV7* ), led to rescue of homologous recombination in *BRCA1*-deficient cells through reinstatement of double-strand-break signalling (Jaspers et al., 2013; Xu et al., 2015). These genes are also downregulated in the human disease, showing their credibility for clinical relevance, while there is some doubt over whether upregulated expression of ATP-binding cassette 1 (ABC1) transporters, also seen in the same mouse models, is a bone fide resistance mechanism in patients (Rottenberg and Borst, 2012; Borst, 2012).

The use of GEMMs in ‘preclinical breast cancer trials’ for new drug combinations is gaining momentum, but as highlighted in the SEARCHBreast workshop, the successful design and execution of appropriate preclinical *in vivo* trials requires close collaboration between clinicians and scientists from the outset. As a proof of concept, a *BRCA1* breast cancer model shown to respond to a combination of cisplatin with PARP inhibition (Rottenberg et al., 2008) with further benefit provided by long-term PARP inhibition (Jaspers et al., 2013), led to the US Food and Drug Administration (FDA) approving such a regime for treatment of platinum-sensitive relapsed ovarian cancer (Ledermann et al., 2012; Matulonis et al., 2016). Disease-specific genetic and syngeneic models (in particular involving transplant of GEMM tissue to recipient strain-matched mice) together with PDX models, hold great potential for the evaluation of patient-relevant regimes. For instance, GEMM-derived and PDX tumours could be used as platforms for preclinical testing of monotherapies versus dual/combination treatment for direct comparison; or testing the merits of neo-adjuvant (first-line) therapy against surgical resection plus adjuvant treatment for efficacy.

### Modelling clinically relevant metastasis in GEMMs

A few key conventional GEMM models (e.g. *MMTV*-PyMT and *MMTV*-*Erbb2*) show manifestation of spontaneously arising metastatic disease in lymph nodes and lungs. These models have been, and continue to be, used to study metastatic disease (Kabeer et al., 2016). So for example by genetically deleting their gene of interest, investigators showed how transforming growth factor beta (*Tgfb1*) and β1-integrin (*Itgb1*) are important in metastasis (Bierie et al., 2008; Huck et al., 2010). Furthermore, conventional GEMMs were instrumental in demonstrating that metastatic spread was an early step in breast cancer progression (Hüsemann et al., 2008). However, as with CDXs and some PDXs described above, an impediment of GEMMs is that relatively few mimic clinical metastasis (i.e. to the brain and bone), which is responsible for the majority of breast-cancer-associated deaths.

A drawback of using conditional GEMMs to study metastatic disease is low penetrance of metastasis. Therefore, large cohorts of animals have to be followed for a prolonged period of time in order for metastatic disease, and effects of therapies thereon, to be accurately measured. While individual GEMM cohorts develop tumours with highly variable latency and inconsistent metastatic penetrance, the syngeneic system used by the Jonkers lab of implanting orthotopic GEMM tumour fragments (Rottenberg et al., 2007) allows a more homogeneous cohort at the study outset. Surgical resection of the mammary tumours in the Jonkers' syngeneic model then permits the development and subsequent evaluation of metastasis, although the authors still report considerable heterogeneity in latency and number of organs affected (Coffelt et al., 2015). This approach could prove useful if it can be extended to other genetic models, although one must consider that *in vivo* transplant/passage of GEMM tissue requires the use of an inbred mouse strain to benefit from immune competency. Propagation of tumours originating from a mixed strain can of course still be performed using immunocompromised recipients to avoid host-graft rejection but this is more costly and limited by a non-physiological microenvironment lacking a functional immune component.

## The impact of technological advances on breast cancer models

The sophisticated models now at our disposal have only been made possible through ongoing technological advancement. It thus seems fitting to chart some of the relevant history as a preface to how we could improve the toolbox to assist in addressing the topical issues of the here and now.

### The imaging revolution

Precise imaging modalities, which are now at the disposal of researchers, have permitted imaging at the single-cell and whole-organism levels. A new era of cancer imaging was heralded with the discovery of green fluorescent protein (GFP) and the realisation that cells could be genetically engineered to express this without detrimental effects (Misteli and Spector, 1997), enabling researchers to locate and track cancer cells and tumour colonies without the need for specific markers or antibodies. This has facilitated mapping of breast tumour development and metastatic progression, as well as evaluation of responses to therapy (Hoffman, 2002). GFP continues to be a useful tool in most breast cancer research laboratories, despite the development of the next generation of imaging probes, allowing easy identification and/or separation of GFP^+^ tumour cells from a mixed population (Lizier et al., 2016; Holen et al., 2015).

Subsequently, the use of bioluminescence allowed sensitive and rapid *in vivo* imaging following injection of the substrate luciferin in animals bearing tumour cells genetically engineered to express firefly luciferase (Edinger et al., 1999). Tumour growth kinetics, as well as therapeutic responses, can now be monitored in the same animal over time (Gross and Piwnica-Worms, 2005; Henriquez et al., 2007). Researchers have embraced this new capability and used it to explore the role of specific molecules in breast cancer development, progression and response to treatment (examples in Serganova et al., 2009; Lu et al., 2010).

Technological advances, such as the use of mammary imaging windows pioneered by the Condeelis and van Rheenen labs (Kedrin et al., 2008), in parallel with the increasing sophistication of molecular probes and other tools have enabled visualisation of tumours *in vivo*. A new generation of confocal and multi-photon microscopes allows visualisation of the interactions between individual breast tumour cells and the microenvironment (e.g. the microvasculature) (Malladi et al., 2016). Initially, this was only possible *ex vivo*, but increased capability for intra-vital microscopy has provided new information regarding how cancer cells move and respond to stimuli in the live animal (Wyckoff et al., 2011; Patsialou et al., 2013; Nakasone et al., 2013). For example, the early stages of local breast cancer invasion (Giampieri et al., 2009) and stromal cell dynamics (Lohela and Werb, 2010; Ewald et al., 2011) have been elucidated using this technology. Furthermore, elegant *in vivo* imaging studies have been used to decipher the behaviour of metastatic cells in real time (Zomer et al., 2015; Harney et al., 2015; Beerling et al., 2016).

Although not widely available, a range of quantitative functional imaging capabilities, e.g. PET (positron emission tomography)/CT, SPECT (single photon emission computed tomography)/CT and PET/MRI (magnetic resonance imaging) are being adapted for preclinical research, leading to increased understanding of tumour development and effects of therapy (Consolino et al., 2016). Furthermore, the advancements in imaging capability have facilitated sophisticated lineage-tracing studies in GEMMs, enabling insights into different cell populations, and in particular the tracking of the mammary stem cell (Rios et al., 2014; van Amerongen, 2015; Sale and Pavelic, 2015) and cancer stem cell (Zomer et al., 2013).

### Improved molecular engineering and the development of targeted genetic models

The application of gene targeting to manipulate the mouse genome (Thomas and Capecchi, 1987; Capecchi, 2005) revolutionised the use of GEMMs to model cancer. Another key advance was the ability to direct these changes to the tissue of interest using site-specific recombinases (e.g. the Cre/*loxP* system, described above) circumventing lethal and off-target effects elicited by germline deletion of fundamental genes (Sauer and Henderson, 1988). This has permitted targeting of breast cancer mutations, such as deletion of key tumour suppressors *BRCA1*, *BRCA2*, *p53* and *PTEN*, specifically to the mouse mammary epithelium to provide models for the human disease (Jonkers et al., 2001; Diaz-Cruz et al., 2010; Melchor et al., 2014). Whilst conditional GEMMs are a key resource, one limitation is the inability to model natural tumour evolution (early versus late events), because of the simultaneous rather than temporal activation of multiple genetic events. The principle of different site-specific recombinases (Olorunniji et al., 2016) to achieve sequential mutations in a time-dependent way is possible, although have not yet been widely applied.

Another major impediment to using GEMMs for dissecting multistage tumourigenesis is the extensive, and time-consuming, breeding programmes required to achieve relevant compound genetic alleles (sometimes requiring in excess of five alleles). Some groups have expedited this process by targeting new alleles in embryonic stem cells derived from pre-existing GEMMs (GEMM-ESCs), an approach that shows excellent potential for streamlined and high-throughput studies (Huijbers et al., 2014, 2015; Henneman et al., 2015). Others meanwhile have applied inducible *in vivo* short hairpin RNA (shRNA)-based knockdown as a means to investigate loss of specific tumour suppressor genes without the need for protracted breeding programmes (Ebbesen et al., 2016).

Within the last decade, increasingly fast, cheap and reproducible genetic sequencing, coupled with improved bioinformatics and pathway analysis tools, has resulted in the identification of mutations that are potentially involved in breast cancer development and progression (Banerji et al., 2012; Ellis et al., 2012; Curtis et al., 2012; Nik-Zainal et al., 2016; Ferrari et al., 2016). Whether these genetic alterations are indeed functional can only be established through experimental validation using *in vivo* tumourigenesis models. High-throughput approaches – such as those using GEMM-ESCs and shRNA – will be necessary if biological verifications of new genes are to match the speed of these discoveries. A recent report, describing how the CRISPR/Cas9 gene-editing system could be combined with GEMM to provide *in vivo* validation of a candidate tumour suppressor in invasive lobular breast cancer demonstrates a powerful new system for the elucidation of functional relevance in the context of mammary carcinomas (Annunziato et al., 2016). This is a perfect example of how advances in one field (gene editing) can be utilised to drive continued development of preclinical modelling in breast cancer. Furthermore, applying high-throughput ‘omics’ and forward genetic screens (e.g. retrovirus or transposons) (McIntyre et al., 2012) to murine-derived tumours provides a platform for discovery of additional cancer-associated mutations (Francis et al., 2015). Of course, these also need to be cross-referenced for relevance in the human disease, but have demonstrable potential to open up new therapeutic avenues; for example, where profiling of mouse tumours highlighted the druggable target *Met* as a secondary mutation in p53-deficient tumours, pointing to a putative new target for treating triple-negative breast cancer (Francis et al., 2015; Pfefferle et al., 2016).

### The expertise in cultivating PDXs provides more clinically relevant models

As discussed above, PDX models have been welcomed by the community as possibly the most promising for precision medicine (Hidalgo et al., 2014; Whittle et al., 2015). Thus, refinement in the protocols to manipulate and propagate primary patient material over recent years (Zhang and Lewis, 2013; DeRose et al., 2013) has been a key advancement. The choice of recipient strains, use of hormone supplementation and site of implant (i.e. intraductal delivery) have all contributed to the success of creating a renewable resource in the form of stably transplantable PDX models that faithfully maintain the characteristics of the original tumour (DeRose et al., 2011). Access to patient samples via clinical interactions or engagement with specialist biobanks such as the Breast Cancer Now Tissue Bank (breastcancertissuebank.org/) is a necessity and the relevant expertise is currently limited to a relatively small number of labs. However, as increasing numbers of breast cancer PDX models are made available through commercial entities (as discussed above) and consortia such as EurOPDX (see Box 1) and the International Breast Cancer Patient-derived Xenograft Consortium (Dobrolecki et al., 2016), enhanced use of these models is expected. In addition, short-term cultures have been generated from some of these and used as models for high-throughput drug screening (Bruna et al., 2016) and to determine mammosphere-forming efficiency *in vitro* (Eyre et al., 2016). These studies illustrate the power of these models, which are likely to be used increasingly in the future. For a comprehensive review of breast cancer PDXs, including their limitations, the reader is referred to Dobrolecki et al. (2016).

## Building a toolkit fit for the future

Although models are continually evolving to incorporate novel technological and biological advances to improve their utility and clinical relevance, there are still refinements to be made. These require tailoring to address the pertinent questions in the field. In this section, we forecast what the future landscape in development of optimised models and tools might be.

### Using GEMMs to model the microenvironment

The importance of the microenvironment in tumour evolution is undisputed (Hanahan and Weinberg, 2011). For example, researchers have demonstrated how macrophages and a highly interactive stroma with cross-talk between the microenvironmental cell types and tumour cells are part of tumour evolution (Qian and Pollard, 2010; Dovas et al., 2013; Noy and Pollard, 2014; Lewis et al., 2016). Furthermore, the immune system can be hijacked into fuelling breast cancer growth and metastasis (Coffelt et al., 2015). Whilst co-injection of stromal and cancer cells to generate CDXs has its place (Orimo et al., 2005), it is time to refine these models. GEMM and syngeneic models are superior for studying the microenvironment as they have an intact immune system, which needs to be given due consideration when testing therapeutic strategies (Coffelt and de Visser, 2015). One caveat of all models is that the microenvironment is murine-derived; even in PDX tumours, the stroma of the mouse host gradually replaces that of the human within 3-4 passages. Regardless, it is noteworthy that studies using GEMMs have elegantly demonstrated just how important the (murine) stroma is for tumour evolution. For example, specific deletion of *Pten* in stromal fibroblasts (Trimboli et al., 2009) or *E2f3* in macrophages (Trikha et al., 2016) modifies tumour growth and metastasis, respectively. Limitations arise, however, because the micro-environmental gene alteration tends to be under Cre/*loxP* control (e.g. *LysCre:E2f3^fl/fl^*), which prohibits application in Cre/*loxP*-driven tumour models. Therefore, syngeneic tumour implants or conventional transgenic tumour models (e.g. *MMTV*-*Erbb2*; *MMTV*-PyMT) are used rather than more physiologically relevant GEMMs. As stromal influences are likely to impact differently on subtypes of breast cancer (Wallace et al., 2011), it is important to consider the use of alternative site-specific recombinases (Olorunniji et al., 2016) to direct the stromal gene modification (e.g. Flp/*Frt*), which would permit investigation with the many available Cre/*loxP* GEMMs.

### Humanisation of models in CDX and PDX models

For many situations – such as preclinical studies using monoclonal antibody therapy, which do not act on the murine gene homologue – there is a desire to use human cells in transplantation models. In order to make *in vivo* models more clinically relevant, researchers have incorporated cells and tissues to increase the human component of the tumour microenvironment i.e. to humanise models. Examples are the implantation of human bone pieces (Kuperwasser et al., 2005; Holen et al., 2015) or tissue-engineered scaffolds seeded with human osteoblasts in immunocompromised animals for studies of bone metastasis (Thibaudeau et al., 2014). The bone discs were either pre-seeded with human tumour cells or tumour cells were injected via the intra-cardiac route once the humanised bone scaffolds were established in the host. These models allow investigation of the interactions between human tumour cells and components of the human bone microenvironment, but they are not yet widely available and standardisation between laboratories is limited by the intrinsic variability in donor bone specimens (Holen et al., 2015).

Furthermore, with the increasing interest in immuno-oncology, efforts have been made to create PDX models that incorporate human immune cells, such as the MiXeno platform from Crown Biosciences. One approach to achieve this is by incorporation of immune cells through co-transplantation of human CD34^+^ hematopoietic stem cells and breast cancer cells in immunodeficient mice (Wege et al., 2011, 2014).

Recently, the Jackson Laboratories described humanised PDX models as the next ‘big thing’ in cancer modelling (www.jax.org/news-and-insights). Indeed, humanised mice in which triple-negative breast cancer PDX responds to immunotherapy are now commercially available. The high cost of these animals is likely to present a barrier to their general uptake, but they represent an important step forward in terms of our ability to investigate the potential utility of immunotherapies in breast cancer.

### Optimising the timelines

A particular challenge for researchers is that breast tumour growth *in vivo* is most often quite rapid (progressing within weeks), in contrast to the longer latency of human disease (involving years and even decades). In this respect, model systems do not represent the clinical reality; however, it is notoriously difficult to work with slow-growing models because results need to be delivered within the normal funding cycle or within the duration of a standard PhD project (3-5 years). As discussed in the workshop, the realisation that ‘slow is better’ is often balanced against the need to know whether a specific genetic or therapeutic manipulation works in a model whilst sufficient time is still left on a particular grant. It was agreed that science funding bodies generally support the use of short-term (and therefore less representative) models, rather than accepting a more limited (but more clinically relevant) output from models where it may take 1-2 years before tumours develop. Many of the workshop participants are members of scientific committees that make decisions on which projects to fund, so there are opportunities to influence thinking in this area with the aim of providing the longer-term funding required to implement the use of more clinically relevant model systems.

### Increasing access to models and material

Working with *in vivo* model systems is expensive, requires expert staff and facilities, and can be time-consuming, presenting a barrier for many researchers. In addition, translation of basic findings into patient benefit requires evidence to be obtained from several model systems. Paradoxically, those who routinely use *in vivo* models are almost always generating more material than they need for the purpose of testing the original hypothesis. The SEARCHBreast initiative (see Box 1) was established in order to bridge this gap by allowing researchers to register the availability of surplus archival material from breast cancer models, providing resources that can be accessed on a collaborative basis, saving time and money (Blyth et al., 2016). Although SEARCHBreast contains material from a range of models, including PDXs, a much larger PDX collection is available through the EurOPDX consortium (europdx.eu/, see Box 1). A rapidly expanding resource, this currently contains 54 luminal, 89 triple-negative and 18 HER2^+^ PDX models, many with associated transcriptomic characterisation; researchers can access these models instead of establishing them *de novo*. With the increasing cost of carrying out large-scale animal studies, we envisage that the sharing of models and material will become the norm, supporting collaborations with laboratories with extensive expertise, with SEARCHBreast as the trailblazer for this type of thinking.

### Developing the models for the future

Breast cancer progression is characterised by the extended period – usually several years – between successful treatment of the primary disease and subsequent relapse (most likely due to the reactivation of dormant disseminated tumour cells in the bone marrow) (Zhang et al., 2013). Model systems that incorporate a dormant phase have been lacking, but some recent studies presented at the workshop have demonstrated that intra-cardiac injection of human tumour cells in mature animals (>12 weeks) shows promise in recapitulating the latency of breast cancer bone metastasis (Ottewell et al., 2014).

As for other models, it was interesting to see one described recently for inflammatory breast cancer, a rare but very aggressive type of breast cancer (Wurth et al., 2015), but there is still a need for a generation of models of male breast cancer and of systems that allow separation of pre- versus post-menopausal disease. We highlight the importance of this point regards disease before and after menopause because recent clinical trials demonstrated therapeutic benefit of bone-targeted agents only in post-menopausal women [Early Breast Cancer Trialists’ Collaborative Group (EBCTCG) et al., 2015]. In addition, new models will be needed as molecular profiling of human breast cancers further stratifies this heterogeneous and complex disease. Better consideration must be given to the specific gene mutations too; for example, genetic modelling of *BRCA1* disease has been achieved predominantly through allelic deletions, but these are not truly representative of the patient-derived pathogenic mutant alleles. The importance of this was recently demonstrated: different *Brca1* mutations elicited similar disease patterns but showed differences in therapeutic response and resistance mechanisms (Drost et al., 2011, 2016). Modelling metastatic disease remains a challenge due to the need to resect the primary tumour to allow sufficient time for metastatic disease to develop, as well as the highly variable number, sites and growth kinetics of resulting metastases. This means that experimental groups need to be large in order to produce meaningful/significant data, which increases the number of animals used in research, puts a burden on resources and can be costly. Furthermore, a flexible study design, such as treatment starting at different time points in different animals, is required for such studies. Finally, the use of models in experimental designs that mimic clinical trials (including in the context of adjuvant therapy) may become more common, suggesting that the clinical relevance of experimental systems will have greater significance in the future.

## Summary and conclusions

*In vivo* models of breast cancer have proven their usefulness in many different contexts and will continue to contribute to our understanding of disease progression, therapeutic response and resistance mechanisms. The field has progressed incredibly in the last 20-30 years and is likely to evolve further with technological advances that enhance the growing arsenal with which researchers can probe unanswered questions. Nonetheless, scientists need to consider the limitations of each model and choose the one that best represents the process they aim to model and addresses their specific research question. New models being developed using patient-derived tumour material have shown promise, but sharing of these resources is very important to allow comparison between studies and make them widely available to the research community. Meanwhile, new and improved models for metastasis and study of the microenvironment are urgently required. In sum, although no perfect *in vivo* model of human breast cancer will ever exist, such models remain valuable research tools complemented by clinical material, *ex vivo* and *in vitro* systems, and we are equipped with the knowledge and technologies to continue improving them.
